# Dating SOS: a systematic and theory-based development of a web-based tailored intervention to prevent dating violence among Brazilian youth

**DOI:** 10.1186/s12889-020-08487-x

**Published:** 2020-03-26

**Authors:** Sheila Giardini Murta, Priscila de Oliveira Parada, Sara da Silva Meneses, João Victor Venâncio Medeiros, Amanda Balbino, Marina Caricatti Rodrigues, Marco Akira Miura, Thiago André Araújo dos Santos, Hein de Vries

**Affiliations:** 1 Department of Clinical Psychology, Institute of Psychology, University of Brasília, Campus Darcy Ribeiro, Brasília, DF CEP 70910-900 Brazil; 2 Department of Design, Institute of Arts, University of Brasília, Brasília, Brazil; 3 Department of Computer Sciences, Institute of Exact Sciences, University of Brasília, Brasília, Brazil; 4 Department of Health Promotion, CAPHRI School for Public Health and Primary Care, Maastricht University, Maastricht, the Netherlands

**Keywords:** Prevention, Domestic violence, Dating violence, Health education, Adolescent health

## Abstract

**Background:**

Dating violence has an alarming prevalence among Brazilian adolescents. School-based preventive programs have been implemented, but remain isolated initiatives with low reach. Health communication strategies based on innovative technologies with a high potential of diffusion are urgent. This study aimed to develop a computer-tailored intervention to prevent victimization and perpetration of dating violence among Brazilian youth.

**Methods:**

The intervention, called Dating SOS (SOS Namoro), is based on the I-Change Model and attachment theory and is a comprehensive preventive program targeted to young people with a current partner. The intervention design included the stages of needs assessment; definition of objectives of change; development of the library of messages; elaboration of a questionnaire for tailoring feedbacks according to the relevant variables; integration of the content in the software Tailor Builder; pre-testing; and usability and efficacy evaluation planning. Dating SOS is composed of four online sessions. The first session gives a tailored orientation on attachment style and risk perception of violence. The second session addresses knowledge on conflict management, positive and negative social models of intimate relationships and an action plan to improve everyday interactions. The third session covers social norms, self-efficacy and an action plan to cope with conflicts. The fourth session discusses attitudes, social support and an action plan to protect from violence. Improvements on the interface and tailoring refinement was done after pre-testing to improve attractiveness and decrease risk of iatrogenic effects.

**Discussion:**

The principal merit of the present study resides in the development of an innovative strategy based on the qualified use of the internet for education surrounding romantic relationships and the prevention of dating violence among adolescent and young Brazilians, a hitherto unaddressed need in the field. The intervention usability and efficacy should be investigated in further studies.

**Trial registration:**

Brazilian Registry of Clinical Trials. RBR-9frj8q. Prospectively registered on July 25, 2019. http://www.ensaiosclinicos.gov.br/rg/RBR-9frj8q/

## Background

Violence received from and practiced against a romantic partner is widespread among Brazilian adolescents. Evidence shows that eight of every ten adolescents (age 15 to 19) have already been victims of or have practiced some type of aggression against an intimate partner at some point in their lives, particularly of a sexual or emotional nature [1]. The predominant constructs of femininity and masculinity contribute to the normalization of dating violence and make its recognition difficult among Brazilian adolescents, as violent manifestations are taken as expressions of love or playfulness inherent to intimacy [2, 3]. This violence invisibility impedes help-seeking and sustains victimization and perpetration of violence by intimate partners in adolescence. Findings of a national study show that adolescents rarely seek help when they are victims of their intimate partner, but when they do so, they prioritize friends and family as the sources while professional help is sought less often [4].

Effective prevention and early intervention services aimed at this type of violence in the adolescent public [5] as well as studies with these purposes are lacking. Results of an evaluation of the implementation quality a law aimed at the eradication, prevention, and punishment of domestic violence against women (the Maria da Penha Law), reveal that educational actions focused on prevention of violence in adolescents, boys or girls, are rare [6]. The paucity of preventive services identified in public policies is also reflected in research. As opposed to other countries in the Americas, which produce frequent research on the prevention of dating violence among adolescents, there is a scarcity of evidence-based prevention interventions in Brazil [7].

Recent Brazilian interventions against partner violence are offered face-to-face in the school environment and conducted in a workshop format by previously trained facilitators [8–12]. Evidences has pointed reduction in sexist and homophobic beliefs [8] and dating violence perpetration [10], increasing in emotion regulation skills [9], and intervention’s acceptability [11, 12]. Even with their promising results, such interventions require institutional support, space in the school’s curriculum schedule, and qualified personnel for their execution in new contexts, all of which hinder their diffusion at a national level resulting in limited public health impact.

An alternative mode of intervention that that may be easier to disseminate and adopted can be computer-tailored intervention (CTI). A CTI consists of the adaptation of health education material to one specific person after an individualized evaluation by an algorithmic process to generate customized feedback and guidance for the user [13]. As in traditional clinical counseling, individual evaluations are followed by feedback and guidance tailored to the participants’ beliefs, behaviors, and needs. However, in contrast to the traditional clinical approach, such feedback and tailored guidance is offered through printed letters, email, mobile phones, and other mobile devices instead of the face-to-face mediation of a professional.

CTI differs from general internet intervention modes (eHealth) that do not use tailored guidance derived from individual evaluations (commonly called computer-based interventions or internet-based interventions) [14]. Several studies of communication in health care reveal consistent evidence that tailored messages, when compared to general messages, tend to be read more often and are remembered and considered more relevant and appealing [15, 16]. Therefore, tailored interventions are more effective and efficient as a health education strategy than other means of general mass communication. Additionally, CTI seems to be highly appropriate for adolescents who are more accustomed to technology than older generations.

While the application of tailored internet-mediated interventions to promote positive health habits is broad [17, 18], their application in the prevention of dating violence is still limited and recent. The first study focusing on this in the literature was conducted by Levesque, Johnson and Prochaska [19], who developed an intervention called *Teen Choices*, based on the transtheoretical model, which sought to prevent dating violence and foster healthy relationships in adolescence. The intervention, composed of a single session, contains different guidance for participants not dating, dating with a low risk of violence, and dating with a high risk of violence. The feedback is tailored according to readiness to engage in healthy relationships and in the seeking and offering of help. The results showed that the *Teen Choices* participants, compared to the control group, presented a significant reduction in perpetration and victimization of physical and emotional violence at 6 and 12 months [20]. The results were more expressive in those who presented a prior history of violence in intimate relationships.

In Brazil, the use of information and communication technologies for mental health promotion is rare [21] and studies using CTI are still embryonic [22] and generally address the reduction of addictive behaviors, such as smoking [23]. Given this situation, the current study innovates by developing an intervention to prevent dating violence among Brazilian adolescents. The intervention, called SOS Namoro (hereafter Dating SOS), was designed to reduce victimization and perpetration of dating violence and increase relationship quality, conflict management skills, and help seeking. It is a universal prevention strategy, targeting youths between 15 and 29, who have an affective-sexual partner at the beginning of the intervention, regardless of being in violent relationship or being exposed to dating violence risk factors. It is based on two theoretical models: the integrated change model, also known as the I-Change Model, and attachment theory.

### Theoretical basis

**Fig. 1:**
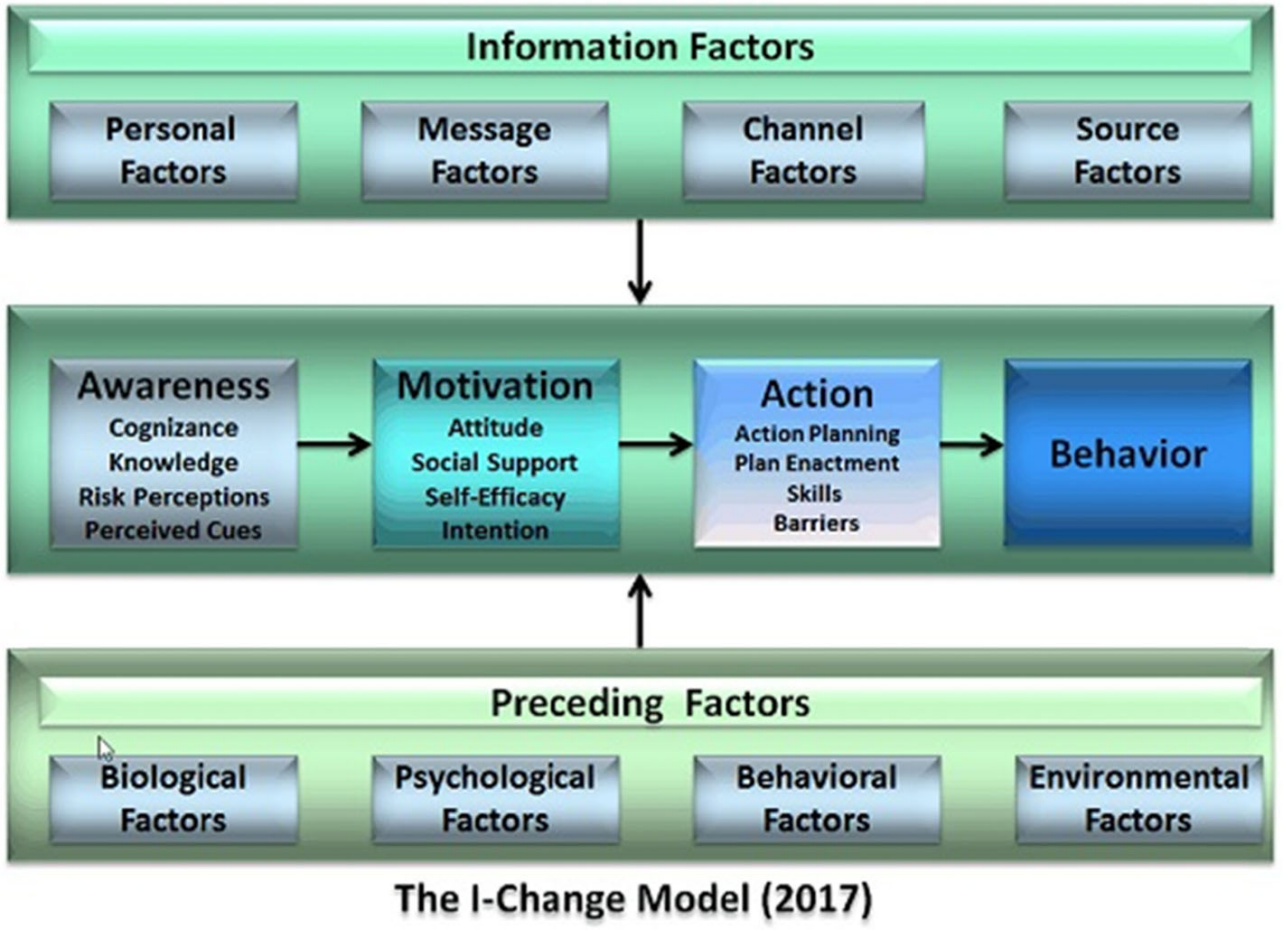
Integrated Change Model (Source: De Vries H. An integrated approach for understanding health behavior; the I-Change Model as an example. Psychol and Behav Sci Intern Journal, 2017;2(2). DOI:10.19080/PBSIJ.2017.02.555585)

The I-Change Model [24, 25] is a composite of several models and socio-cognitive theories, including Azjen’s theory of planned behavior [26], Bandura’s social cognitive theory [27], Prochaska’s transtheoretical model [28], Janz and Becker’s health beliefs model [29], and goal setting theories [30]. According to the model (see Fig. 1), behavior and behavior change is a process of several phases and each phase is determined by different types of factors.

First of all, a person needs to become aware of the problem, which is determined by awareness of his/her own behavior (i.e. is the behavior that I am doing appropriate, am I behaving in a violent way), knowledge (i.e. what are considered to be sexually violent behaviors?); risk perceptions (i.e. am I at risk of violating someone and what are the consequences); and perceived cues (i.e. what are the situations that may prompt me to become sexually violent). Second, when a person becomes aware of a problem and its own role in the problem, he/she may become open to become motivated to change unhealthy behavior. Factors that are important here are: attitudes towards the behavior and the perceived pros and cons of it (e.g. when I am sexually violent I can show that I am in charge of things), the perceived social support (e.g. my mates think it is okay to be sexually violent), and self-efficacy (e.g. when I feel stressed I find it difficult to control myself and not become sexually violent). The combination of positive attitudes, supportive norms and high self-efficacy will lead to a positive intention to perform a healthy behavior. Third, a positive intention does not automatically translate into the desired goal behavior but requires action planning (e.g. what will I do when I feel stressed), actual realization of these plans, the skills, and sometimes the removal of certain barriers.

Lastly, the I-Change Model postulates that these factors are influenced by a wide array of predisposing factors, such as psychological, behavioral, socio-environmental factors. Additionnaly, the model postulates that awareness, motivation and action can be influenced by developing communicative actions, of which the effects are determined by personal factors (e.g. level of education), message factors (e.g. clearly written with clear instructions, tailored to a specific person), source factors (e.g. using peers that provide information), and channel factors (e.g. the choice for interpersonal, personal or eHealth channels).

Dating SOS will operate through three mechanisms outlined by the I-Change Model. The first mechanism consists in raising awareness of dating violence as a problem by promoting knowledge of the phenomenon and its impact on health. The second comprises strengthening motivation to protect oneself against violence from an intimate partner by increasing attitudes favorable to the non-violent management of conflicts and unfavorable to the acceptance of violence, enhancing critical thinking about pro-violence social norms, aiding in the identification of healthy models of loving relationships and sources of social support when facing violence, and developing self-efficacy to deepen emotional intimacy in the relationship and reduce the use and acceptance of violent conflict resolution strategies. The third covers fostering self-protective intentions by means of developing clear action plans for facilitating day-to-day communication with the partner, managing conflicts without violence, and protecting oneself should violence arise (for example, seeking help). Applications of the I-Change Model to study and intervene on violence, showed the importance of these determinants [31], as well as the efficacy of intervening on these determinants to reduce violence successfully [32, 33].

In addition to the I-Change Model, attachment theory was adopted as a secondary theoretical basis for Dating SOS [34]. Although this theory has been extensively used to explain how attachment may explain the process leading to intimate partner violence [35] and prevention to dating violence [36], its use in CTI to this end has not yet been recorded. The choice of attachment theory to base Dating SOS upon resides in the evidence that adolescents with insecure affective bonding styles tend to use more destructive conflict management strategies and are more frequently victims of dating violence than those securely attached [37, 38]. Evidence from a study of a Brazilian sample with couples in various stages of romantic relationships corroborates these findings [39].

Insecure attachment styles stem from rejective, inconsistent, intrusive, or abusive parenting practices that foment the development of internal working models characterized by distrust and insecurity in the establishment of loving bonds as well as an image of themselves as undeserving of love [40, 41]. In turn, secure attachment style results from parental care responsive to the needs of the child who, as a consequence, develops lifelong internal working models predictive of a positive image of oneself and one’s partner, facilitating the building of intimate relationships in adulthood and increasing the likelihood that they be longer-lived, healthier, and more secure. In this way, romantic love in adolescence and adulthood can be understood as an attachment process stemming from the first attachment experiences in childhood.

Low levels of anxiety and avoidance in affective relationships characterize secure attachment style, whereas insecure attachment styles present elevated levels of anxiety and/or avoidance [42]. Avoidance is related to the degree of comfort individuals experience in the face of emotional closeness and intimacy. Applied to romantic relationships, highly-avoidant individuals are reluctant to establish intimate relationships and seek emotional comfort based on the concept that intimacy is threatening and the seeking of emotional support undesirable and its offer impossible. Anxiety denotes the degree to which individuals worry about being rejected or abandoned by their romantic partners. High levels of anxiety manifest themselves in vigilance for signs of partner distancing as well as elevated investment in maintaining closeness by compulsively searching for support, which can overwhelm partners [40, 43] and translate into violent outbursts in addition to indifference, threats, insults, and possessiveness. Secure attachment is a protective factor for the quality of romantic relationships [44], while insecure attachment is one of the risk factors for dating violence, contributing to deficient emotional regulation [41] and conflict resolution abilities [38, 39].

In line with attachment theory, it is hoped that Dating SOS can broaden the knowledge of its participants regarding their own attachment styles and how their anxiety levels and avoidance affect their romantic relationships. In due course, this should boost action plans for emotion-regulating, including understanding aspects of the relationship that evoke negative emotions (such as exposure to violence, its perpetration, or both) as well as awareness of one’s own emotions and managing how they are expressed. It is expected that, upon receiving guidance tailored to their own attachment style and to the levels of dating violence experienced, the participants will recognize opportunities for implementing action plans more responsive to their needs and those of their partners. This in turn should reduce the use of negative conflict resolution strategies, such as distancing and excessive control, and augment the use of positive ones, such as offering to help and emotional closeness – depending on the dynamics of the relationship (violent or not) – thereby improving the quality of the romantic relationship and reducing victimization and perpetration of violence against one’s partner. Finally, one anticipates that the intervention can boost action plans and skills for seeking help or ending a relationship for those participants exposed to violence as opposed to tolerating violence, deflecting them from strategies intended to cope with insecure attachment and instead leading them to strategies related to a secure attachment style.

This article is intended describe the development process of Dating SOS, a computer tailored intervention grounded in theory for preventing dating violence and improving the romantic relationship quality of Brazilian youths. It is anticipated that the study can advance the production of knowledge in the field in three ways. First, it adopts dating violence as the focus, something little done thus far in computer tailored interventions. Second, it bases its intervention design on a collection of empirical studies of dating violence among young Brazilians, a population largely ignored in preventive actions and unstudied in this area. Third it selects, in an original way, theoretical models yet untested in CTI for dating violence prevention.

## Method

### Intervention

SOS Dating is an online intervention in which the participants receive tailored guidance based on their attachment style, their experience of having been involved in a romantic relationship with or without violence, their knowledge of dating violence, their attitudes towards dating violence, their positive and negative relationship models derived from those they spend their time and live with, the social norms regarding dating violence in their social circles, their sources of social support available for coping with violence, their self-efficacy for coping with conflicts nonviolently, and their action plans for improving the day-to-day quality of their romantic relationship and for managing conflict and protecting against violence. The tailoring also take in account the participants’ gender, sexual orientation and status of violence. Status of violence in the romantic relationship ranges from an absence of violence through episodes of violence to regular violence. In cases of regular violence or episodes of violence the direction (victimization, perpetration, bidirectional) is also considered.

The intervention comprises four individual sessions, available at http://sosnamoro.geppsvida.com.br. The content, objectives, and variables used in the customization are described on Table 1. Each session lasts approximately 15 minutes and occurs 1 week after the previous one. The feedback and participant guidance customizations were made using the *TailorBuilder* software, used in CTIs already described in literature [45, 46].

**Table 1:** Content and objectives of the SOS Dating

	Content	Objectives
Session 1	Knowledge of attachment style	Increase knowledge of attachment styles and their impact on romantic relationships.
	Violence risk perception	Boost risk perception regarding violence experienced in a romantic relationship.
Session 2	Knowledge of violence, relationship quality, and conflict management	Identify types of dating violence: physical, psychological, sexual, and material.
		Recognize the characteristics that classify relationships as safe and gratifying as opposed to insecure and violent.
		Differentiate negative dating conflict management strategies from positive ones and its relation with attachment style.
	Positive and negative models of loving relationships	Identify models of violent loving relationships: friends, aunts and uncles, cousins, parents, grandparents, stepparents, and neighbors
		Identify models of positive loving relationships: friends, aunts and uncles, cousins, parents, grandparents, stepparents, and neighbors
	Action plans for increasing relationship quality in day-to-day coexistence	Identify damage potential in risky action plans for daily interaction: lack of communication, ignoring the partner’s needs, shutting down and ignoring one’s own needs, and not respecting the partner’s individuality.
		Construct positive action plans for daily interactions: noting the partner’s needs and providing help sensitively, sharing personal needs with the partner, asking for help, and respecting the partner’s individuality.
Session 3	Social norms regarding dating violence	Weaken the acceptance of socially disused gender stereotypes that justify violence.
	Self-efficacy in handling dating conflicts	Recognize the most challenging dating conflict situations to deal with in a non-violent way: cheating, lying, provocation, an attack by one’s partner, a bad temper, being drunk, and repeated violence.
	Action plans for non-violent dating conflicts	Identify the potential for damage in risky action plans for handling conflict: retaliation, resignation, use of alcohol or drugs, withdrawal, empathy (for victimization).
		Construct positive action plans for handing conflict: regulation of emotions, assertiveness, recognizing errors and apologizing, empathy (for perpetration), and ending the relationship.
Session 4	Attitudes toward dating violence	Strengthen the understanding that violence can be detrimental by harming the relationship, the victim, the perpetrator, and future generations.
		Weaken the perception that violence can be advantageous by granting power, self-defense, affectional gains, and financial gains.
	Social support for protecting oneself from dating violence	Recognize sources of social support for protection in the face of relationship violence.
		Actively seek social support and give clear indications that support is needed, as people may be reluctant to be invasive and help.
		Think critically and refuse social support for accepting violent relationships on the basis that they are a source of economic support, wealth, power, or that one should honor the relationship or protect the children.
	Action plans for freeing oneself from dating violence	Construct positive action plans for protecting oneself against violence: separating from the partner, seeking help, and ending the relationship.

In Session 1, called “My Relationship,” data from the attachment style and dating violence scales are used to generate tailored feedback. Based on the answers given to those scales, the participants get immediate feedback about their attachment style (secure or insecure/evasive, insecure/anxious or insecure/preoccupied), the degree of violence in the relationship (no violence, episodes of violence, or high levels of violence), and the direction of violence (victimization, perpetration, or mutual).

In Sessions 2 to 4, the participants initially answer a questionnaire focused on the dating violence determinants from the I-Change Model, including knowledge, social modeling for violent or positive intimate relations, action plans to improve the day-to-day relationship quality (Session 2 – “Improving Coexistence”), social norms, self-efficacy, action plans for conflict management (Session 3 – “Dealing with conflicts”), attitudes, social support, and action plans for self-protection in the face of violence (Session 4 – “Protecting Yourself Against Violence”). After this, they receive tailored feedback, as in Session 1.

In each session, opening and concluding narratives were used to help the participants identify with the intervention. The feedback is offered via text passages, available as narratives or lists of items, and self-monitoring exercises. They are posted to a website in a password-protected area and sent via e-mail as a PDF attachment as well. Email reminders are sent to participants 1 week after every session, if they have not completed the previous session or started the next one. All interactions between the participant and SOS Dating happen online, without the mediation of a human facilitator.

### Procedures

The development of SOS Dating comprised eight steps: needs evaluation, definition of the intervention’s objective, construction of the customization questionnaire, elaboration of the message library, entering the content into the customization software, intervention pre-test, and evaluation planning [47].

#### Step 1: Needs evaluation

The needs evaluation consisted of three empirical studies with youths, both victims and not of dating violence, to identify relevant objectives for the intervention and develop its content.

The first study had the purpose to examine the psychosocial determinants of dating violence and was conducted with seven focus groups of adolescents (*n* = 12) coming from public and private schools and young students (*n* = 16) from private and public universities. The focus groups explored the psychosocial determinants of dating violence, as recommended by the I-Change Model, and were conducted separately according to gender and school/university. The data, examined by deductive thematic analysis, was categorized as knowledge, social norms, social support, models, attitudes, self-efficacy, action plans, and suggestions for preventive initiatives against dating violence. The results pointed to gaps in the knowledge of the psychological and material manifestations of dating violence, with physical and sexual being the most acknowledged forms. Similarly, ignorance of non-violent conflict resolution among students was apparent, as well as what positive and healthy aspects of romantic relationships would entail. Dating violence was reported as acceptable in several situations, such as in the case of betrayal, when attacked first, and if one or both partners are intoxicated. Pro-violence social norms attached to sexist stereotypes were commonly reported. The participants reported financial dependence and having kids as conditions that can promote submission to violence and inhibit social support for leaving the abusive relationship. Finally, they reported action plans with potentially negative impacts, such as resigning themselves to violence, acting indifferently to the partner, and responding to violence with violence, but there were some positive plans as well, such as terminating the relationship or looking for professional help. The participants suggested that preventive interventions might develop critical thinking about gender stereotypes and foster respect for the partner’s individuality.

The second study investigated the characteristics, triggers and consequences of violent conflicts in dating relationships. It was conducted via individual interviews with ten participants, six men and four women, aged 17 to 27, who had experienced dating violence, either as victims or as perpetrator-victims (i.e. bidirectional violence). The results were analyzed using deductive thematic analysis. The participants described their relationships as a mix of positive and negative experiences, with strong affective involvement but with the use of ineffective strategies of conflict management. Among those, resignation to the partner’s impositions was reported; threats, possessiveness, and casting blame to control the other’s behavior; poor emotional control (cursing and bouts of rage) accompanied by psychological, physical, and mental aggressions; attempts at reconciliation and repairing; and the withdrawal into silence and indifference toward the partner. It was reported that all these strategies resulted in new conflicts and subsequent future violence, causing great suffering to the parties involved. Furthermore, data indicated the hyperactivation of the system of attachment in the face of signals interpreted as abandonment by the partner, for example silence and distancing accompanied by anxious responses, such as emotional outbursts to make the other listen.

The third study, aimed to examine emotional intimacy and safety in the romantic relationship, keeping attachment theory as the theoretical lens. It used semi-structured individual interviews with youths between sixteen and twenty-three years of age who had dating experience. The results revealed variability among the interviewees regarding the intimacy experience, with higher levels of intimacy being connected to the exchange of social support between partners and sensitivity to each other’s needs, and threatening levels of intimacy connected to evasiveness when seeking support from the partner and negative conflict resolution strategies. The data aligned with the recommendations of attachment theory, which are that partner’s availability and accessibility are the central path for promoting emotional closeness in romantic relationships.

In short, the needs evaluation demonstrated the urgency of the intervention, taking into account its main findings: violence is seen as natural in romantic relationships; gender stereotypes justifies attitudes of acceptance of violence; negative social modeling for romantic relationships are largely present in closer relationships; psychological and material violence are barely recognized as violence; betrayal is the most difficult situation to not using violence against the partner; there are negative strategies to cope with conflicts, including submission, emotional dysregulation and withdrawal; and violent conflicts are related to non responsive and hostile partners’ answers.

#### Step 2: Defining the target group and intervention objectives

The data collected from all three needs evaluation studies underpinned the elaboration of the intervention’s objectives (Table 1) and target group. The target group selected for the intervention was youths (15–29 years old) who had a romantic partner. The results of needs assessment suggested that the intervention should (a) prescribe objectives to address psychosocial determinants of dating violence which were also in line with socio-cognitive ecological models such as the I-Change Model, (b) address not only violence but relationship quality too, focusing on the partners’ mechanisms of availability and accessibility to strengthen emotional closeness, as informed by attachment theory, (c) discuss gender stereotypes and how they are connected to attitudes and social norms that sustain violence, (d) consider attachment styles and their impacts on emotional regulation as a barrier to self-efficacy for coping with conflict and breaking the connection with the partner, and (e) consider contextual diversity in action plans, ranging from daily interactions, to conflict situations as well as currently being in a violent situation.

#### Step 3: Construction of questionnaire for tailoring

In order to address the various objectives to be addressed in a computer tailored intervention, questionnaire based on the I-Change Model was developed, titled the Dating Violence Determinant Questionnaire. The instrument considers several determinants predicted in the I-Change Model: awareness (eight items, for instance, “Conflict can occur in a relationship without there being violence”); attitudes (six items, for example, “When you depend on the partner’s money to live, it’s better to endure the violence”); social norms (nine items, for instance, “Someone who cheats deserves to be attacked”); models (two items, for example, “Of the people you know, who has a romantic relationship (dating or marriage) that is positive, with companionship, respect, and affection? You can choose more than one option: parents, aunts and uncles, grandparents, friends, neighbors, nobody”); social support (one item, “Who would you ask for help to get out of a violent relationship? You can choose more than one option: father, mother, siblings, friends, church group, police, nobody”); self-efficacy (eight items, for example, “How hard would it be for you to ‘keep cool’ and not be violent towards your partner if he or she had lied to you”); and action plans, subdivided into action plans to help in daily life with the partner (six items, for instance, “If I was down, I would tell him/her my problems and ask for help”), dealing with conflicts (14 items, for instance, “I’d get my revenge, I’d get back at him/her”), and protecting against violence (seven items, for example, “I would end the relationship”).

We have chosen a closed question format with multiple choice answers for the following determinants: awareness, social support and action plans. A Likert scale was used to evaluate the determinants: attitude (4 points, from disagree completely to agree completely), social norms (5 points, from almost everyone to almost no one), and self-efficacy (4 points, from very easy to very difficult). The instrument was submitted to semantic validation; confusing terms or those with double meanings were corrected.

#### Step 4: Elaboration of the message library

Once the intervention objectives had been established and the Dating Violence Determinant Questionnaire had been created, the elaboration of a repository of advice messages was started, resulting in a message library. The message library was developed according to recommendations of Kreuter et al. [47]. First, tables were built for each determinant predicted in the I-Change Model (awareness, attitudes, social support, social norms, models, self-efficacy, and positive and negative action plans) with the message title (for identification purposes), objective (changes targeted by that message), origin (which items in the questionnaire supplied the data that is the basis of that message), format (didactic resource for message expression, such as narration, vignette, comparative table, video, etc.), theory and base evidence (studies that support the message’s content), main problem (principal presumed life context of user), tone (emotional aspect of the message, i.e., empathetic or informative), concepts (message content), and message (the guidance itself).

After this systematic planning, the messages were elaborated by psychology students, who were told to write freely, as if they were giving advice to a friend. This strategy was adopted to maximize the accessibility and friendly tone of the messages. Next, the messages were reviewed by researchers with expertise in the research subject. At this stage, it was verified that the elaborated guidance covered the objectives and concepts expected of it.

This process resulted in 67 messages. In session 1, four variations were created based on attachment style and eight based on the status of violence in the current relationship. In session 2, eleven messages were elaborated regarding awareness of dating violence, six about violent relationship models, six about non-violent relationship models, and lastly four action plans were created to cover daily life with the partner. In session 3, six message variations were developed about social norms, three on self-efficacy, and four of action plans for conflict management. Finally, in session 4, six messages were composed concerning attitudes, six about social support, and three for action plans to deal with dating violence. Table 1 shows the objectives of each session and the customization variables by session.

The messages consist of a combination of text segments, with the presence and position of each in the presented message body determined by the participant’s answers. The answers to the items are used as input, generating a final classification of attachment style. This classification becomes the variable responsible for determining which segments are used in the message subsequently presented to the participant. Moreover, it is possible to use this previously calculated variable as input for the succeeding questionnaires.

Care was taken that the combination of various messages would result in coherent texts. The text segments were constant only within a specific set of classifications, serving as introductory, connecting, or concluding passages, whereas the remaining part was tailored based on the response of the participant to the question.

The Portuguese language requires special attention to grammatical gender. To write a text that seems reader-directed, it is necessary to use the correct gender for the participant and partner. Since gender use occurs according to well-defined rules, it was possible to adapt the text for several possibilities.

#### Step 5: Entering the content into the software for tailoring

In parallel with the previous step, the intervention’s webpage and its visual identity were developed. After finishing the message library, all instruments were entered into *TailorBuilder*, which then customized the guidance to the participants’ profiles. This stage involved the employment of a programmer and a UX (user experience) expert to conceive and implement the interaction with the SOS Dating participants.

#### Step 6: Intervention pre-testing

Next, the intervention’s pre-testing was performed. Three pre-testing sessions were held, the first with three young people potential users, the second with three professionals working at centers for victims of domestic violence, and the third with twenty university students. The main findings of pre-testing were related to the format and content:

- The young people potential users thought the messages were too long and that SOS Dating’s interface was not clear;
- The professionals noted a risk for side-effects in the guidance regarding the bidirectional violence and conflict management for participants whose relationships were already violent.
- All participants reported perceiving the intervention as relevant.
- Responding to these findings, an extensive review was performed of the intervention’s visual aspects and content.
- A new visual identity, aiming for greater interface elements clarity, was proposed and content readability was improved.
- The messages were shortened to reduce the cognitive demand on the participants;
- Tailoring adjustments were made in conflict management and actions plans regarding the status of violence in the relationship, with the purpose of preventing iatrogenic effects and boosting participant identification with the intervention.

After these adjustments, new wave of pre-testing was done with two potential users and two experts in domestic violence, which approved the aforementioned changes.

#### Step 7: Evaluation planning

Two evaluation modes were planned, efficacy and usability.

### Efficacy evaluation

#### Objectives

The intervention was designed to reduce victimization and perpetration of physical, sexual, and psychological violence in dating (primary outcomes) and to boost the quality of the romantic relationship as well as conflict management skills and help seeking (secondary outcomes).

#### Calculation of the sample size

The calculation of the sample size for this study uses a conservative procedure to identify a 10% reduction in dating violence prevalence on the intervention group compared to the control group. Adopting a significance level of 5%, a testing power of 80%, and a 50% dropout rate over the course of the study, it was calculated that each condition should have 610 individuals.

#### Participants and design

An experimental design with a pre-test evaluation and three-month follow-up will be used. Youths between 15 and 29 years of age and their current partners will be invited to participate. A sample of 1220 youths, which will be randomly and evenly divided between the experimental and control groups, is expected. Participants younger than 15 and older than 29 or not currently in a dating relationship will be excluded from the intervention.

The intervention group will receive the SOS Dating intervention, while the control group will receive general information about dating violence, life skills, and sexual and reproductive rights, based on the book “Telling a night out from a trap: a Guide for the Empowerment of Teens in Intimate Relationships” (Diferenciando Baladas de Ciladas: um Guia para o Empoderamento de Adolescentes em Relacionamentos Íntimos) [48], available at http://www.geppsvida.com.br/publicacoes/cartilhas-e-guias/.

#### Randomization

The randomization for the experimental and control group will be performed after the eligibility evaluation, the express agreement to participate in the intervention through the consent form, and the baseline evaluation. The placement into one group or the other will be performed automatically by the *TailorBuilder* software, as has been done before in other similar interventions [45, 46].

#### Recruitment

Several strategies will be used to advertise SOS Dating and recruiting participants: (a) posting on the social networks of social movements related to fighting violence against women, LGBT rights, and youth engagement; (b) advertising on e-mail lists of research associations and research groups interested in related topics; (c) informing the Federal District Ministry of Education, which is connected to public schools frequented by the target audience; and (d) communicating it to student health and guidance services of Brazilian public universities.

#### Instruments and procedures

##### Eligibility evaluation

**Fig. 2:**
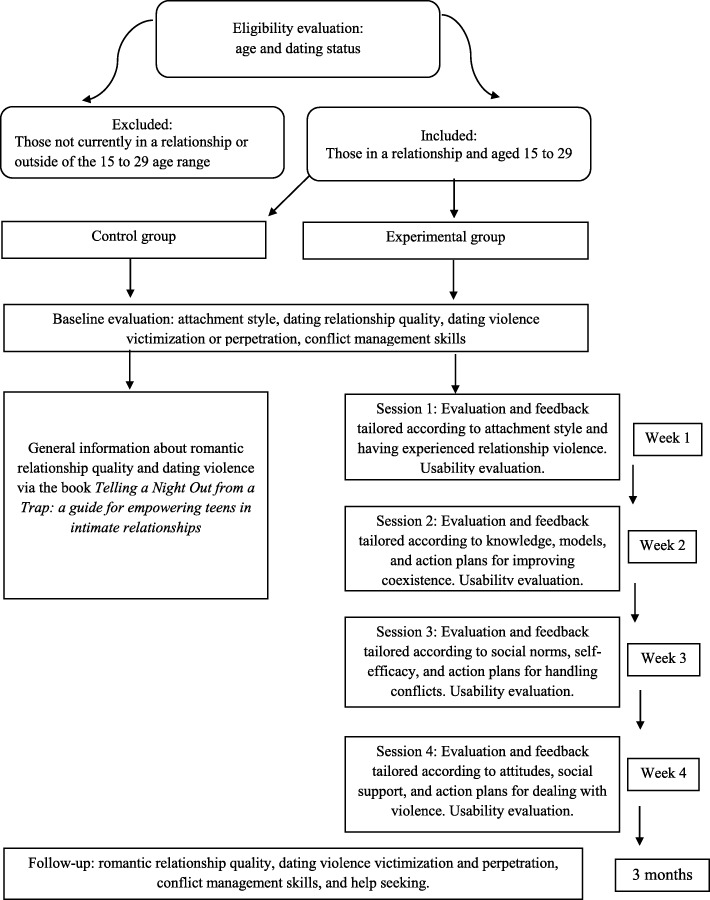
Evaluation flowchart

After clarifications regarding the research and declaration of voluntary participation via the consent form, the participants will answer a social-demographic survey composed of closed questions about age, gender, level of education, socio-economic level, religion, sexual orientation, parents’ professions, parents’ educations, previous dating experience, romantic status (whether single or dating), and how long have they been dating. The participants between 15 and 29 years of age who have a current romantic partner will be included in the intervention, while those who do not meet both criteria will be excluded (Fig. 2).

##### Baseline

The included participants will next respond to the baseline evaluation, which will assess attachment style; conflict resolution skills; victimization and perpetration of psychological, physical, and/or sexual violence in their partners; and relationship quality.

### Attachment

Attachment will be evaluated by the Experience in Close Relationships Scale, short version (ECR-Short). The original scale comprises twelve items and was developed by Wei et al. [49] from the full Experience in Close Relationships Scale, which has 36 items, by Brennan, Clark and Shaver [50]. In Brazil, the adaptation was done by Natividade and Shiramizu [51], and contains 10 items that evaluate two factors: anxiety related to attachment (preoccupation with maintaining the romantic relationship and the partner’s responsiveness and the need for physical and emotional closeness, for example: “I need a lot of assurance that I am loved by my partner”) and avoidance related to attachment (discomfort with emotional proximity, with dependence on a romantic partner, and a preference for emotional detachment, for example: “In general, I try to avoid strong affective closeness with my partner”). Low avoidance and low anxiety indicate secure attachment, while high rates of avoidance and/or anxiety reveal insecure attachment styles. The items are answered on a seven-point Likert scale. Higher scores indicate higher levels of anxiety and avoidance and thus an insecure attachment style.

### Conflict resolution skills

Conflict resolution skills are evaluated through the Conflict Resolution Behavior Questionnaire (CRBQ), developed by Rubenstein and Feldman [52] and adapted to Brazil by Delatorre and Wagner [53]. The instrument is composed of 22 items that evaluate the frequency with which strategies of conflict resolution by avoidance (for example: “I stay cool and distant and don’t pay attention to the other person”), attack (for instance: “I am sarcastic”), and agreement (for instance: “I apologize to the other person”) are employed. A five-point Likert scale is used, ranging from 1 (never) to 5 (always). In the study by Delatorre and Wagner [53], internal consistency levels of 0.68 to 0.79 were found.

### Victimization and perpetration of dating violence

The Conflict in Adolescent Dating Relationship Inventory (CADRI) [54], Brazilian version by Oliveira et al. [1] will be used to evaluate victimization and perpetration of dating violence. In this study, a short version of CADRI will be used. This version is composed of 24 items that evaluate perpetration and victimization various types of violence: psychological (16 items, for instance: “He/she talked to me in a hostile or mean way”); physical (four items, for example: “He/she pushed me or he shook me”); and sexual (4 items, for instance: “I touched him/her sexually when he/she did not want to be”). The participants are requested to indicate the frequency of violent events in the current relationship on a four-point scale (always = it happened six or more times in my current relationship, sometimes = it happened three to five times in my current relationship, rarely = it happened once or twice in my current relationship, never = it has never happened in my current relationship).

### Relationship quality

A short version of the instrument *Romantic Relationship Quality Scale*, developed by Murta et al. [55], will be used, with 21 items on a five-point Likert scale (1 = nothing to do with us; 5 = everything to do with us). The scale is composed of two factors. The first is sexual-affective closeness (alpha = 0.87), comprising 12 items (for instance “I’m affectionate with him/her”), which evaluates mutual help and support, appreciation of each other’s company, self-revelation, empathetic listening, respecting the other’s world (including his/her family), and enjoying physical and sexual intimacy with the other person. The second factor is responsiveness (alpha = 0.88), comprising 9 items (for example “I trust him/her”), which evaluate trust in the other person, admiration for the other person, the perception of what the other person likes or feels, commitment to the relationship, and acceptance of the other’s individuality.

#### Follow-up

At three months after the end of the intervention, the experimental and control group participants will again respond to measurements of conflict resolution skills, victimization and perpetration of violence, and relationship quality. Additionally, they will also fill out the Seeking Help Questionnaire. This instrument, developed by the authors, is composed of three closed questions that inquire whether the respondents sought any sort of help to deal with their affective relationship since they first entered the research website, whom they asked for help, and what the perceived efficacy of the help received was.

Additionally, the Dating SOS participants will be asked to evaluate the impact of the intervention by responding to the Dating SOS Impact Questionnaire, composed of one closed question (How would you evaluate the effects of Dating SOS in your life?) with a multiple choice answer (e.g.: It helped me to end the violent relationship) and three open questions (If Dating SOS made no difference in your life, what do you attribute this to?; If Dating SOS had some effect in your life, what do you attribute this to?; Comment freely about what you thought of Dating SOS).

### Usability evaluation

With the aim of evaluating the attractiveness of each session and identifying aspects for possible improvement, a usability evaluation will be done at the conclusion of each session of the intervention.

A questionnaire with both open and closed questions, which evaluate the satisfaction of the user with the guidance received and interaction with the tool will be used. Six closed questions investigate satisfaction with the theme of the session, questions, guidance, navigation, quantity of information, and errors. Three open questions examine satisfaction with the layout, guidance content, and suggestions for improvement. Finally, the user is asked which aspect of the Dating SOS was most attractive and which was the least attractive.

### Statistical analysis

Descriptive statistics will be used to describe the characteristics of the participants in the baseline and usability data. The principal analyses will consist of intra- and intergroup comparisons between the baseline and the three-month follow-up on the outcomes of victimization and perpetration of physical, psychological, and sexual violence; quality of the romantic relationship; and conflict management skills. Multivariate analysis of covariance will be used to examine the efficacy of the intervention on these outcomes. The attachment style and the relationship’s violence status will be used as covariates. In addition to this, intergroup comparisons will be conducted using the *t*-test on the three-month follow-up outcome of seeking help. Moderation analysis will be employed to evaluate the differential effects of the intervention based on gender, age, and sexual orientation. A significance level of 0.05 will be adopted.

Omitted or incomplete data will be handled via multiple imputation using the Missing Value Analysis procedure of SPSS Statistics. Intention-to-treat analyses will be carried out, and all randomized participants will be posteriorly included regardless of having completed the intervention or not. The effects of Dating SOS will be estimated utilizing Cohen’s d (where values around 0.2 = small effect size; around 0.5 = medium effect size; and around 0.8 = large effect size).

### Ethical aspects

This study was approved by the Committee for Ethics in the Human and Social Sciences of the University of Brasília (approval CAAE 71749317.9.0000.5540).

## Discussion

We believe the principal merit of the present study resides in the development of an innovative strategy based on the qualified use of the internet for education surrounding romantic relationships and the prevention of dating violence among adolescent and young Brazilians, a hitherto unaddressed need in the field. It is hoped that studies based on Dating SOS and the intervention itself will serve, to one extent or another, to strengthen professional practices in prevention and psychotherapy as applied to dating violence, supported by a more nuanced understanding of the phenomenon.

The primary limitation of this study lays in data collection in the pre-testing stage from youths from Brasília, who could be distant in culture and sociodemographic characteristics from their counterparts in other regions of Brazil. Given the vastness of the country and its cultural diversity, it is possible this would result in a loss of cultural sensitivity for the intervention when implemented with youths in small cities, rural areas, and traditional communities, such as indigenous ones. It is also possible that the omission of an evaluation of partner mutability, that is, how influenceable the partner is or is not by the efforts of the intervention user constitutes an important limitation. This could be a target of future studies.

Should there be positive evidence for the intervention’s usability and efficacy, it will become a useful tool for bringing scientific knowledge to adolescents and will contribute to the debate around the broadening of educational services and early interventions that integrate the scope of public policies directed at dealing with violence against women and the promotion of mental health in adolescents of both genders in Brazil. Should the evidence be insufficient or negative regarding the intervention’s efficacy, new studies should correct problems, remedy gaps, and undertake additional testing.

In conclusion, dating violence has an elevated prevalence among Brazilian adolescents. School-based preventive programs have been implemented, but remain isolated initiatives with insufficient reach. Health communication strategies based on scalable technologies are compelling. Taking this into account, we developed Dating SOS, an innovative computer tailored intervention designed to prevent dating violence and improve the quality of affective-sexual relationship among Brazilian adolescents. Its production was based on clear theoretical basis and empirical evidences derived from Brazilian context. The romantic partner changeability and a broader geographic diversity sample should be considered in the design of similar technologies. The usability and efficacy of Dating SOS should be examined in future studies.

## Data Availability

Data are available from the corresponding author on reasonable request.

## Abbreviations

CTI: Computer-tailored intervention
I-Change Model: Integrated change model
UX: User experience
ECR-short: Experience in Close Relationships Scale, short version
CRBQ: Conflict Resolution Behavior Questionnaire
CADRI: Conflict in Adolescent Dating Relationship Inventory
